# Research on the impact of irregular structure group construction on rail transit bridge piers and its response to wind load

**DOI:** 10.1038/s41598-023-37717-6

**Published:** 2023-06-28

**Authors:** Yan Wang, Zheng Chen, Yi Qi

**Affiliations:** 1 School of Civil Engineering, Qingdao University of Technology, Qingdao, 266520 Shandong China; 2 Guangxi Key Laboratory of Disaster Prevention and Engineering Safety, Guangxi University, Nanning, 530004 Guangxi China; 3 Beijing Urban Construction Group Co., Ltd, Beijing, 100088 China

**Keywords:** Civil engineering, Environmental impact

## Abstract

The possibility of deformation and collision in existing railway bridge foundation structures due to the construction of a group of large irregular structures in close proximity and their potential for overturning under strong wind loads is posed as a potential threat. The impact of the construction of large irregular sculptures on bridge piers and their response under strong wind loads is primarily investigated in this study. A modeling method based on real 3D spatial information of the bridge structure, geological structure, and sculpture structure is proposed to accurately reflect their spatial relationships. Finite difference method is employed to analyze the impact of sculpture structure construction on pier deformations and ground settlement. The bridge structure exhibits small overall deformation, with maximum horizontal and vertical displacements of the piers located at the edge of the bent cap on the side of the critical neighboring bridge pier *J24* adjacent to the sculpture. A fluid–solid coupling model of the interaction between the sculpture structure and wind loads with two different direction is established using computational fluid dynamics, and theoretical analysis and numerical calculations are conducted on the sculpture's anti-overturning performance. The internal force indicators such as displacement, stress, and moment of the sculpture structure in the flow field under two working conditions are studied, and comparative analysis of typical structures is conducted. It is shown that sculpture *A* and *B* have different unfavorable wind directions and specific internal force distributions and response patterns due to the influence of size effects. Under both working conditions, the sculpture structure remains safe and stable.

## Introduction

With the rapid development of China's economy, the demand for transportation infrastructure has increased. To provide fast traffic links between suburbs, urban rail transit is an ideal choice. According to statistics, a total of 50 cities in mainland China have put into operation 9192 km of urban rail transit lines, of which there are over 950 km of elevated lines^1^. Since elevated railway lines often cross or are parallel to urban roads, it is inevitable that rail transit bridges will be affected during the municipal construction of urban roads. The control standards of railway elevated bridges are strict, and the allowable deformation is small, so it is necessary to strictly control construction quality when constructing under railway bridges^2, 3^. For example, when constructing shallow-buried pile foundation structures close to rail transit bridges, special consideration should be given to the deformation effects of structures on bridge pile foundations, pile caps, piers, U-beams, rails, and other basic structures, as well as to their resistance to strong wind loads^4^. Therefore, it is necessary to study the impact of construction under rail transit bridges.

Scholars have performed substantial research on the deformation law of railway infrastructure under the influence of external loads. Feng et al.^5^ used analytical methods to study the mapping relationship between the vertical deformation of bridge structures and the deformation of high-speed railway rails and proposed a corresponding analytical model. The rail deformation under three typical bridge structural deformations was calculated by using analytical and finite element numerical methods, and the evolution of the rail geometry in this case was analyzed. The results show that the mapping coefficient between the bridge structure deformation and the rail deformation increases nonlinearly with increasing bridge structure deformation amplitude. Gou et al.^6^ provided a method to quantitatively evaluate track deformation due to bridge deformations and interlayer degradation. Using this method, real-time state assessment of high-speed railway tracks was carried out on the basis of real-time monitoring of bridge deformation. The analysis and research were validated against the 3D finite element model and were used to study the impact of key parameters. Salcher et al.^7^ evaluated the influence of soil-structure interactions on the dynamics of railway bridges based on numerical simulations of natural frequencies, natural vibration modes and equivalent damping coefficients. Based on the energy-variational principle, a coupling vibration analysis model of a high-speed railway simply supported beam bridge-track structure system was established by Jiang et al.^8^ under the consideration of the effect of shear deformation. The ANSYS and MIDAS finite element numerical calculation methods were compared with the analytical methods established in that paper. The analysis method established in the study was used to evaluate the natural vibration characteristics of the structural system under different interlayer stiffnesses and different rail lengths of subgrade sections. Han et al.^9^ developed an integrated system that includes a global positioning system (GPS), accelerometer and anemometer to obtain the responses of a long-span bridge to extreme wind loadings. An adaptive recursive least squares filter was adopted to separate the slow-varying movements, and the total displacement with enhanced measurement accuracy was obtained from the combined quasi-static and high-frequency dynamic displacements. The results show that the proposed technique can significantly improve the accuracy of displacement measurements under strong winds.

Regarding the analytical solution of the structural deformation of bridge piers, many studies have drawn a large number of useful conclusions^10–14^. Bimschas et al.^15, 16^ developed a reliable mechanical model to describe the complete distribution of flexural deformations along the member and at all loading levels. This paper presented the theoretical concept of inelastic bending deformation analysis of reinforced concrete piers. When the discontinuous stress field was used to determine the string force, the influence of oblique cracks related to shear on the bending deformation behavior was considered. The mechanical method of inelastic bending analysis of reinforced concrete piers introduced in a supporting paper was applied, and the results were compared with the experimental measurement results of two large-scale experiments. Chen et al.^17, 18^ proposed a method to analyze the influence of the settlement of multiple piers on a train–track–bridge coupled dynamic system. The mapping relationship between the pier settlement and rail deformation is derived theoretically. Based on the train–track–bridge dynamic interaction theory, a detailed train–track–bridge dynamic model was established. The field test results were verified by using this model to calculate the dynamic response of the train–rail–bridge system under different pier settlement values.

Similarly, laboratory experiments and numerical simulations have been widely used in the analysis of bridge structural deformation problems^19, 20^. A detailed 3D finite element model was developed by Liu^21^ to resolve the interactions among the pier column, pile foundation, surrounding soil, and heavy truck loading to facilitate analysis. After validating the model using the general analysis M method, the developed model was used to study the behavior of the bridge under the combined effects of temporary road loads. Zheng et al.^22^ presented a numerical investigation of the deformation response of geosynthetic-reinforced soil (GRS) mini-piers under service load conditions. Simulation results from a parametric study indicate that the backfill soil friction angle, backfill soil cohesion, reinforcement vertical spacing, and reinforcement stiffness have the most significant effects on the settlements and lateral facing displacements of GRS mini-piers under service load conditions. Zhang et al.^23^ used the finite element code LS-DYNA to numerically simulate collisions between rockfalls and bridge piers to study impact forces, bridge response, and track structural deformation. A two-step numerical modeling technique was proposed to determine the operational safety index of high-speed trains.

At the same time, there have been many studies on the structural response of buildings under wind loads^24–27^. In contrast, there have been relatively few studies on the anti-overturning performance of structures such as municipal facilities under strong wind loads. Zuo et al.^28^ conducted a three-phase experiment to study the wind loading of sign structures with rectangular sign faces. Five models of representative rectangular signs of different configurations were tested in a wind tunnel. The results revealed the significant influence of the geometrical configuration of rectangular sign structures on the wind load. Tse et al.^29^ carried out numerical research on four typical configurations of noise mitigation structures and their characteristics with regard to wind loads. The turbulence model and model parameters, equilibrium atmospheric boundary layer modeling, grid discretization, etc., were considered in the numerical model to improve the numerical accuracy. One configuration was also numerically validated using wind tunnel test data. Solari^30^ summarized the research on wind loads in structural wind engineering and discussed the analytical, experimental and numerical tools commonly used to ensure structural safety.

From the above studies, it is clear that most of the research on the deformation of bridge structures under the influence of external loads has been focused on the effects of vibration loads, such as earthquake loads and train operating loads. However, there have been few studies regarding the influence of the construction of shallow-buried pile foundation structures under existing rail transit bridges on structural deformation. In particular, when the size of a structure is large, the shape is irregular, and the distance from the pier column is relatively unfavorable, the structure is at risk of overturning under the action of a strong wind load. Once overturned, it will cause irreversible damage to bridge structures such as piers, which will seriously affect the safety of trains.

At present, the construction of large structures under railway bridges at very close distances is rare. There are also no detailed studies on this situation. In particular, the sculpture structure in the project example studied here is extremely irregular, as it is a group combination of large-scale irregular shallow-buried pile foundation structures. The construction of the sculpture structure under the railway bridge will cause certain deformation effects on the bridge piers, thereby affecting the track stability. In addition, since the project site is located in the offshore sea wind-affected area, according to meteorological data, the average maximum wind force reaches level 9 gale, so the wind load will have a great impact on the sculpture structure itself during the construction process and after the construction is completed. Because the overturning of the structure group will cause damage to the bridge piers, under various unfavorable conditions, the research on this project has practical significance and implications for innovation.

Therefore, it is necessary to study the construction of shallow-buried pile foundation structures under existing railway viaducts and the deformation of the viaduct structures under the influence of wind loading. Additionally, studies of the deformation of rail transit bridges under the influence of the construction of shallow-buried pile foundation structures and the anti-overturning performance of structures under the effect of a strong wind load are needed. In terms of research methods, although most relevant studies also used numerical simulation methods, their modeling was often limited by the numerical simulation software itself, and it is impossible to represent the structure very realistically. In this paper, refined 3D solid modeling is performed as the preprocessing step of the numerical simulation, which overcomes the oversimplification of numerical modeling and restores the size parameters, spatial position, construction process and material parameters of the engineering object itself to the maximum extent, improving the numerical calculation accuracy.

## Engineering background overview

Qingdao Subway Line 11 has a total length of approximately 58 km, and the entire line is mainly in the form of an elevated bridge, with a maximum design speed of 120 km/h. Below the railway bridge, a new urban road will be built, and the road and bridge will intersect at approximately orthogonally. According to the plan, a group of municipal sculptures will be built at the intersection of the road and the bridge, directly below the U-beams, namely, sculpture *A*, sculpture *B* and sculpture *C*.

**Figure 1:**
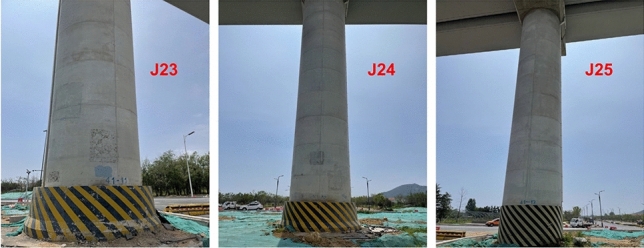
Condition of the piers.

According to the construction location of the sculptures, the section of the rail transit bridge is K41 + 243.125 ~ K41 + 296.125, and the pier numbers are *J23*–*J25*. Prestressed concrete U-beams are adopted, of which the span between piers *J23* and *J24* is 28 m and the span between piers *J24* and *J25* is 25 m. The concrete grade of the beam is C55, the piers and the pile caps are made of C40 concrete, and the bent cap is made of C50 concrete. Figure 1 shows the pile foundations in the elevated bridge section within the influence scope of the sculpture construction. The status of the pier is shown in Table 1.

**Table 1:** Condition of the pile foundation.

Pier number	Pier size (m)	Pile cap size (m)	Pile diameter (m)	Pile length (m)	Number of piles	Pile spacing (m)	Pile bearing stratum
J23	3.9 × 3.6	7 × 11.5 × 2.5	1.5	34	6	4.5	Moderately weathered granite
J24	3.9 × 3.6	7 × 11.5 × 2.5	1.5	32	6	4.5	Moderately weathered granite
J25	3.9 × 3.6	7 × 11.5 × 2.5	1.5	35	6	4.5	Moderately weathered granite

The sculptures are models made through special-shaped casting. Sculpture *A* has a maximum height of 16 m, a maximum span of 12 m, and an overall length of 18.3 m. Sculpture *B* has a maximum height of 9 m, a maximum span of 7.6 m, and an overall length of 14.3 m. Sculpture *C* has a maximum height of 4 m, a maximum span of 3.29 m, and an overall length of 8.3 m. Each sculpture is supported by a strip foundation, with a C40 reinforced concrete structure forming the main body of the foundation.

**Figure 2:**
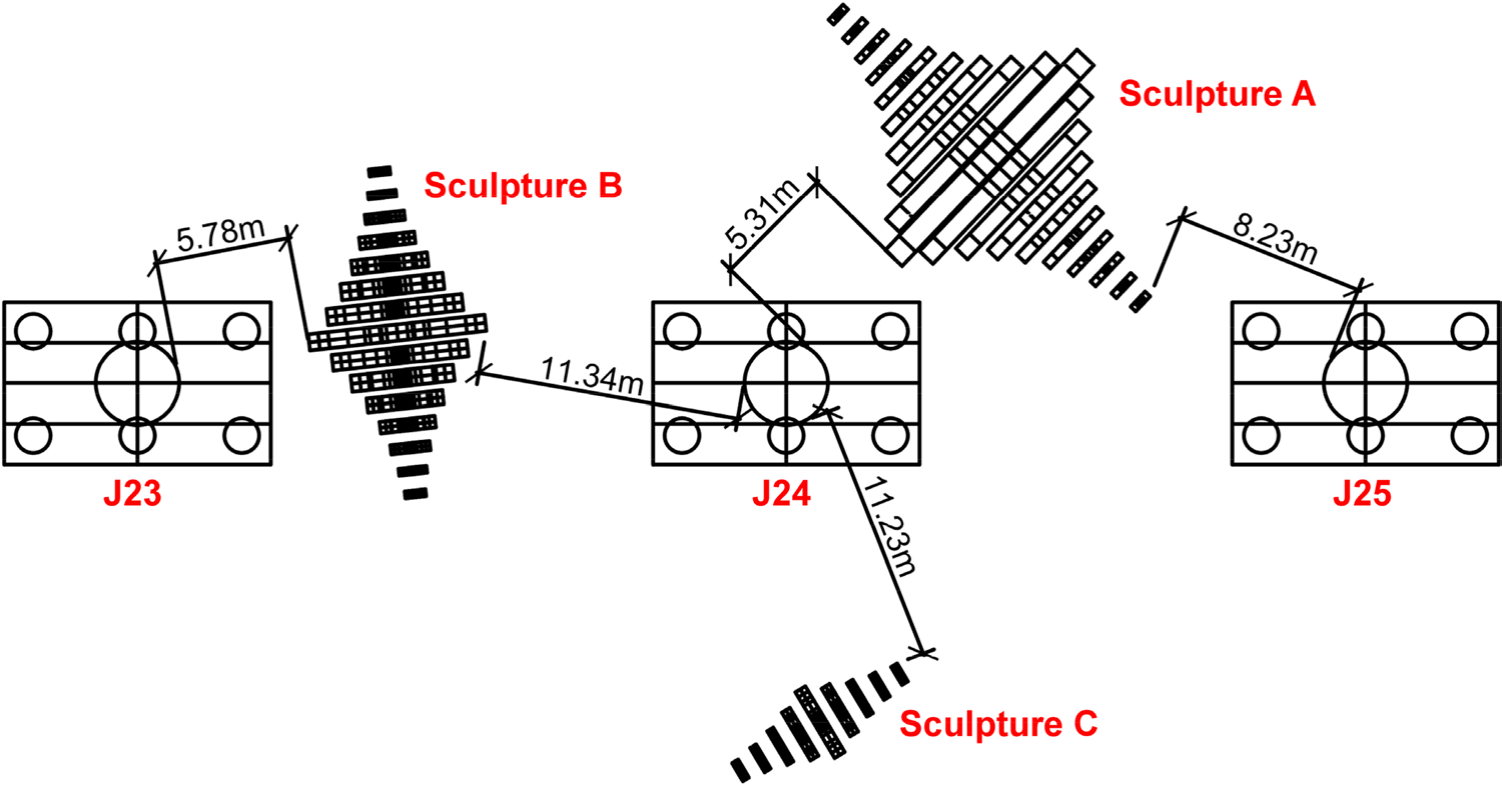
Lateral distances between the sculptures and piers.

Some of sculptures *A* and* B* are located within the vertical projection range of the rail transit bridge, and sculpture *C* is completely outside the projection range of the bridge. Sculpture *B* is approximately perpendicular to the bridge, and the line connecting the bases of sculptures *A* and *C* obliquely crosses the bridge. The vertical heights of the three sculptures are all lower than the heights of the bridge bent caps and U-beams, and the bases are located outside the vertical range of the piers and pile caps of *J23*, *J24*, and *J25*. The distance between the sculptures and the piers and U-beams is shown in and Fig. 2.

## 3D solid modeling

To reflect the spatial positional relationship between different structures, a 3D solid space model of the sculpture structure, bridge structure and stratum structure is established. This 3D solid model will be used for numerical calculations in subsequent analysis of the effects of sculpture construction and wind loads. According to the design documents, field investigation and geological condition data, Rhino software is used for modeling to realize the digital twin simulation of the solid structure.

**Figure 3:**
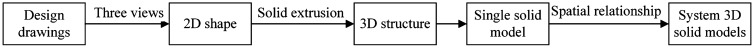
Modeling process.

The modeling process in *Rhino* software heavily relies on detailed parameters from the three views. Therefore, during the modeling process, it is necessary to establish corresponding solid models and composite models for the sculpture structure, bridge structure, and geological strata based on the planar parameters of their respective front, side, and top views, as well as their spatial positional relationships. Firstly, the outer and inner contours of the structures are determined based on their front views, defining the two-dimensional shapes in the plane. Using the side view, the dimensions and thickness of the structures are determined, and the 3D structures are created using the solid extrusion feature in *Rhino* software. By considering the top view and the spatial relationships between the structures, the individual solid models are placed in their respective positions within the spatial coordinate system. Finally, after completing the modeling of all structures, the overall model of the system is obtained. The modeling process is illustrated in Fig. 3.

### 3D solid model of the sculptures

**Figure 4:**
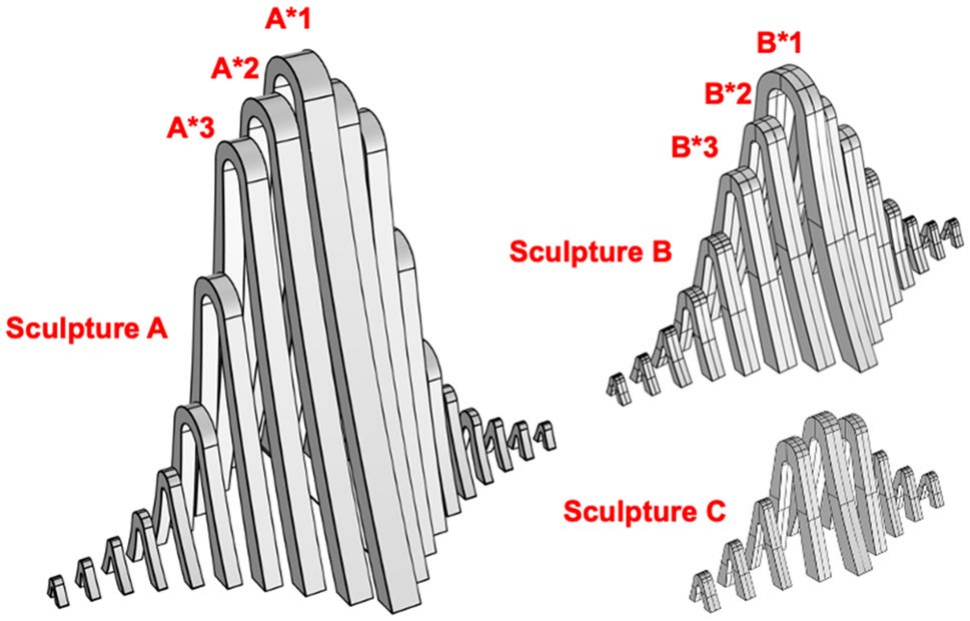
3D solid model of the sculptures.

According to the design drawing and construction drawing of the sculpture structure, each component of the sculpture is a special-shaped structure, the top and bottom are not of equal width, and the overall shape is narrow at the top and wide at the bottom. The established 3D solid models of sculptures *A*, *B*, and *C* are shown in Fig. 4.

### 3D solid model of the bridge and strata

According to the bridge design documents, parameters such as the type, elevation and size of the corresponding U-beams, bent caps, pile caps, and pile foundations of piers *J23*, *J24* and *J25* are determined, as shown in Table 2.

**Table 2:** Dimensional parameters of the bridge structures.

Size type	Pier number		
	J23	J24	J25
Back span (m)	28	25	30
Beam height (m)	0.4	0.4	0.4
Bent cap size (m)	10.67 × 4.4 × 1.7	10.67 × 4.4 × 1.7	10.67 × 4.4 × 1.7
Bent cap height (m)	24.5	24.5	24.5
Pier size (m)	3.9 × 3.6 × 24.0	3.9 × 3.6 × 24.0	3.9 × 3.6 × 24.0
Pile cap size (m)	7.0 × 11.5 × 2.5	7.0 × 11.5 × 2.5	7.0 × 11.5 × 2.5
Pile diameter (m)	Φ1.5	Φ1.5	Φ1.5
Pile length (m)	34	32	35
Pile number	6	6	6
Pile spacing (m)	4.5 × 4.5	4.5 × 4.5	4.5 × 4.5

**Figure 5:**
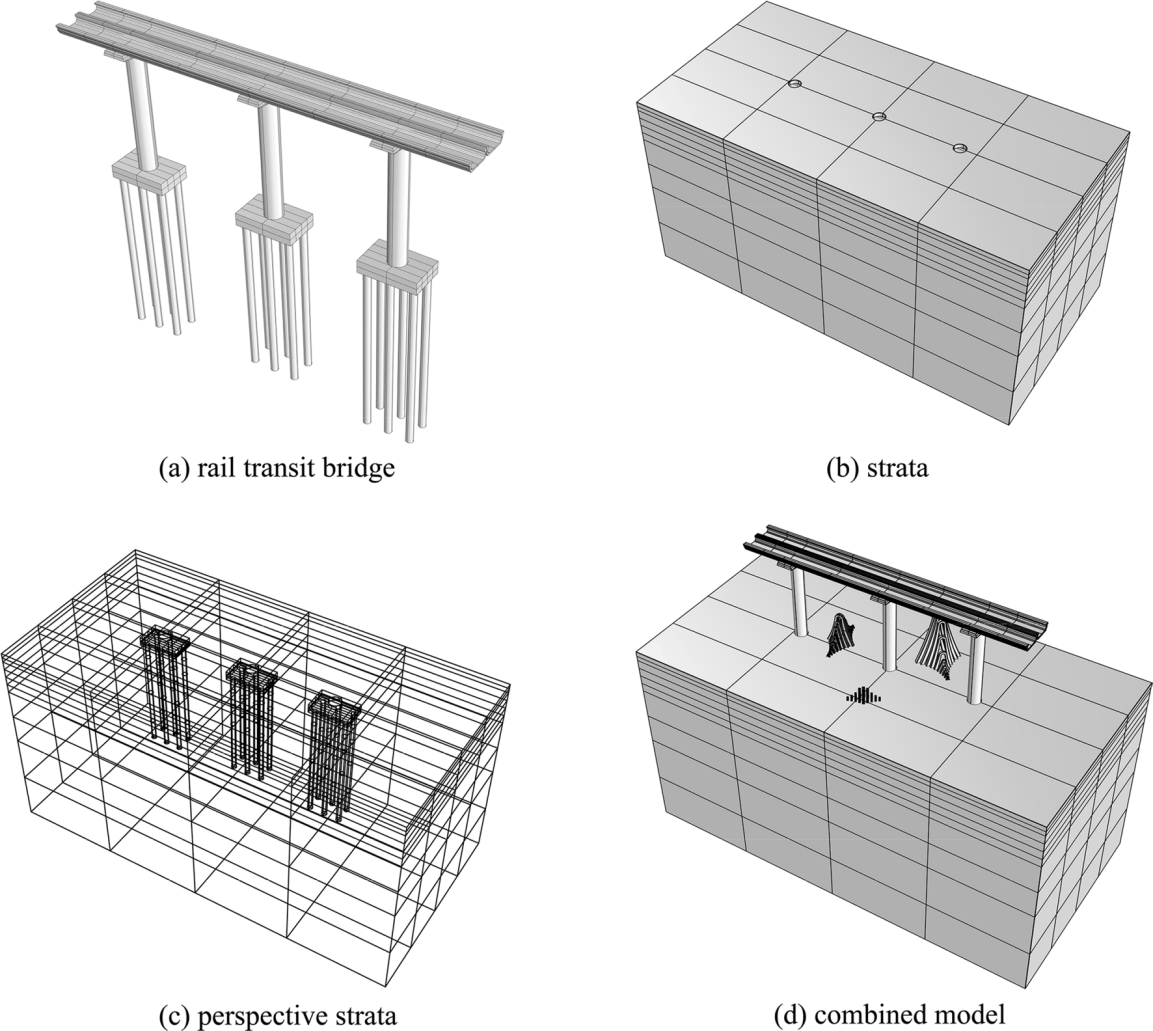
3D solid models.

There are 5 kinds of strata encountered in this project; from top to bottom, they are plain fill, silty clay, medium-coarse sand, strongly weathered granite and moderately weathered granite, with thicknesses of 2.8 m, 5.2 m, 5.2 m, 18 m and 28.8 m, respectively. Considering the intersection of the stratum and the bridge structure, it is necessary to perform Boolean operations to delete structures such as piers, pile caps, and bridge piles in the strata. The 3D solid model of the sculptures, the rail transit bridge, and the strata are combined. According to the planned placement of the sculptures, a combined 3D solid space model is obtained. The 3D solid models are shown in Fig. 5.

## Influence of sculpture construction on the safety of rail transit bridges

### Construction of the numerical model

**Figure 6:**
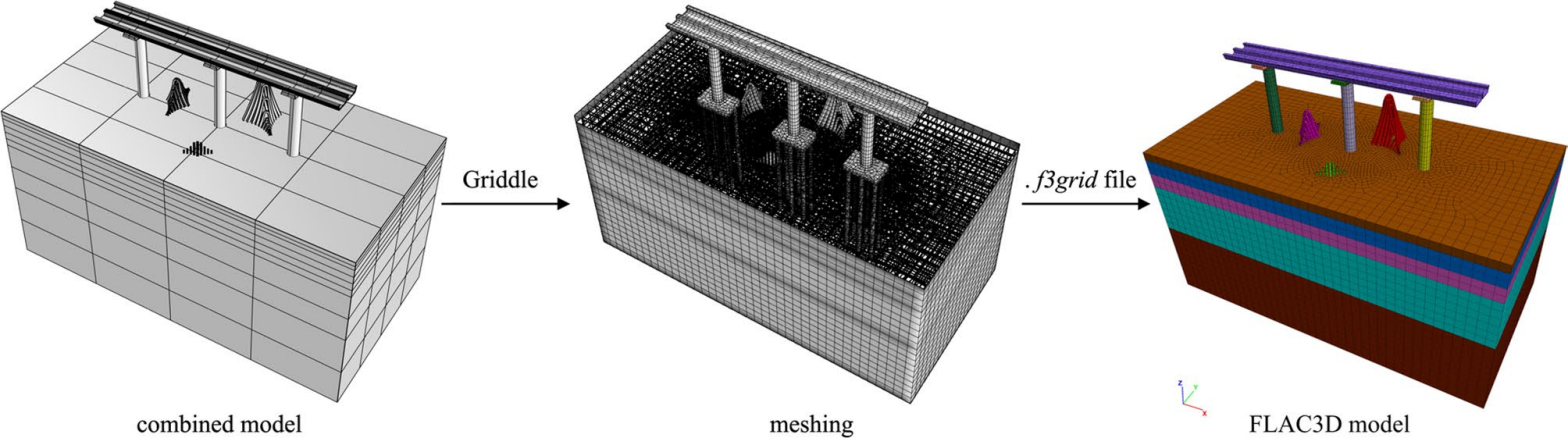
Import process.

According to the combined 3D solid model and the specific placement of the sculptures, a finite difference method (FDM) numerical simulation using *FLAC3D* software is carried out for piers *J23*, *J24* and *J25* within the influence range. In order to import the 3D solid models created in the previous section into *FLAC3D* software, the *Griddle* plugin is utilized to generate model meshes within *Rhino* software. The generated mesh files were exported as *.f3grid* files and imported into *FLAC3D* software. The models were then reorganized for subsequent numerical calculations. The import process is illustrated in Fig. 6. The structural deformation of the bridge caused by the construction of sculptures *A*, *B*, and *C* is calculated.

**Figure 7:**
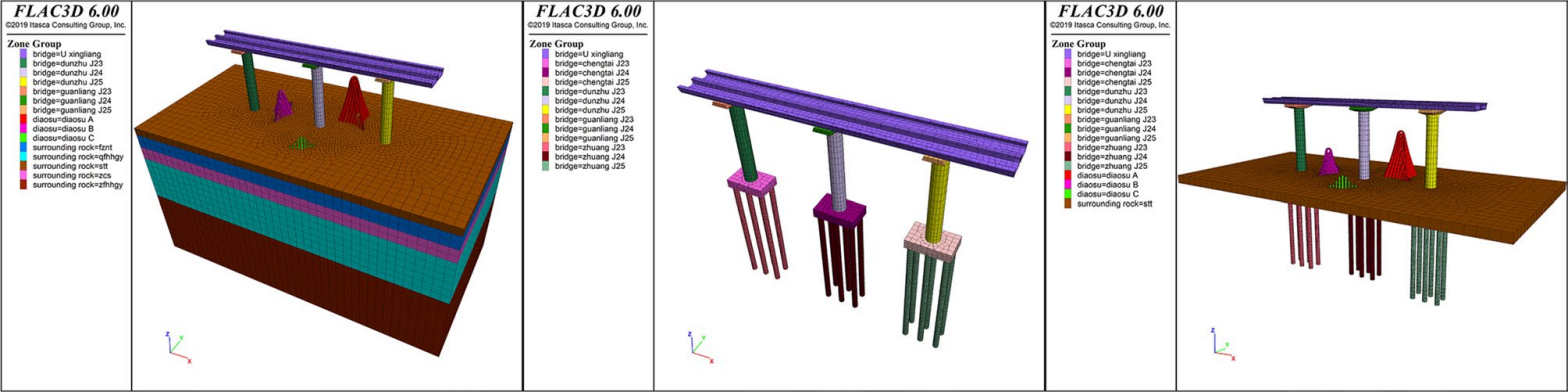
Numerical model.

Due to the randomness and complexity of the physical and mechanical properties of geotechnical materials, in the modeling and calculation process, the main factors are considered, the secondary factors are ignored, and the specific problems are appropriately simplified. The following assumptions are adopted in this numerical simulation: (1) the surrounding rock and soil materials are homogeneous and isotropic continuous media; (2) the influence of tectonic stress is not considered in the initial stress field simulation. To eliminate boundary effects, the model boundary dimensions are set to 2–4 times the size of the core research area. The primary focus is the core area composed of three piers and three sculpture structures, with a planar range of approximately 55 m × 25 m. Taking into account the depth of the pile foundations, the *x* × *y* × *z* dimensions of the model is set to 120 m × 60 m × 80 m. The boundary constraints are set to be free for the top surface and a fixed displacement for other surfaces. That is, there is no limit to the displacement of the top surface in the three coordinate directions*.* The displacement of the bottom surface is fixed in all three coordinate directions. The surrounding boundary has a fixed displacement boundary condition in the* x*-direction and* y*-direction; only *z*-direction displacement is allowed. The *Mohr‒Coulomb* model is used for each stratum, and the *Elastic* model is used for each bridge structure and sculpture structure. The origin of the coordinate system is located at the left boundary of the model. The positive *x*-axis is arranged along the centerline of the bridge, the *y*-axis is perpendicular to the centerline of the bridge, and the positive *z*-axis is vertically upward. The model material parameters are shown in Table 3. The numerical model used in the simulation analysis is shown in Fig. 7.

**Table 3:** Model material parameters.

Strata number	Strata name	Thickness *h* (m)	Volumetric weight *γ* (kN/m^3^)	*C* (kPa)	*φ* (°)	Elastic modulus *E* (MPa)	Poisson's ratio *υ*
1	Plain fill	2.8	18	10	20	10.5	0.30
7	Silty clay	5.2	19.4	24	10	20	0.35
9	Medium-coarse sand	5.2	18.5	0	38	30	0.30
16	Strongly weathered granite	18	22	600	45	45	0.22
17	Moderately weathered granite	28.8	24	1300	55	12,000	0.15
	C40 concrete		24			26,000	0.2
	C50 concrete		24.5			27,600	0.2
	C55 concrete		25			28,400	0.2
	Q235 steel					210,000	0.3

### Analysis of the calculation results

#### Deformation of the piers

**Figure 8:**
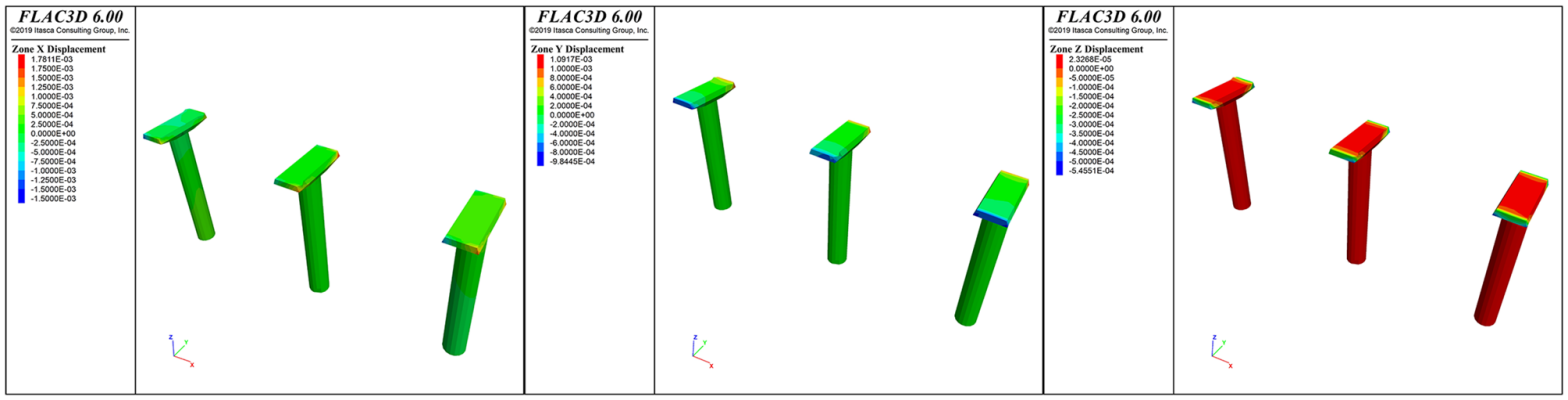
Displacement of the bridge piers.

According to the numerical simulation calculation results, after the sculpture is constructed, the maximum displacement of the bridge piers in the *x*-direction is approximately 1.78 mm, which occurs at the edge of the bent cap of pier *J24* near the side of the sculpture. The maximum displacement of the bridge piers in the *y*-direction is approximately 1.09 mm, which occurs at the edge of the bent cap of *J24* near the side of the sculpture. The maximum displacement of the bridge piers in the *z*-direction is approximately 0.54 mm, which occurs at the edge of the pier of *J24* near the side of the sculpture. The displacement of the bridge piers is shown in Fig. 8.

#### Ground settlement

**Figure 9:**
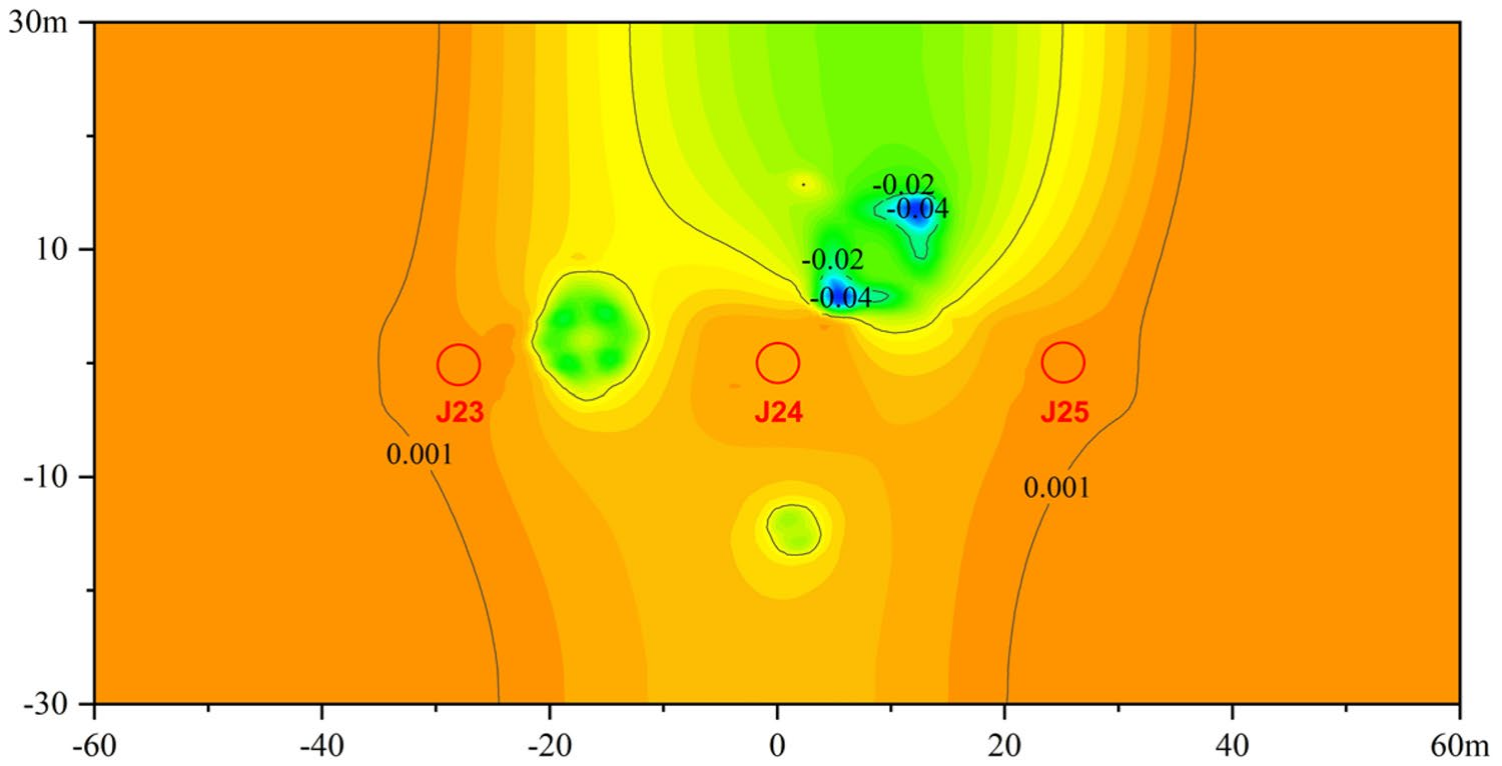
Ground settlement.

Due to the large self-weight of the sculpture structure, ground settlement will occur in the surface soil due to the influence of its construction. According to the numerical simulation results, the ground settlement of the top stratum of plain fill is shown in Fig. 9. Within the scope of the model plane, the placement of the sculpture produces a significant vertical settlement. Among them, the position where the base of sculpture *A* is in contact with the soil has the largest settlement, which is approximately 0.04 m. Since the construction of sculptures *A* and *B* has a large influence range on the deformation of the surrounding soil layer, the influence range overlaps, forming a saddle-shaped settlement tank.

Affected by sculpture construction, the maximum horizontal displacement of the bridge pier structure is approximately 1.78 mm, which occurs at the edge of the bent cap of pier *J24* near the side of the sculpture, and the maximum vertical displacement is approximately 0.54 mm, which occurs at the edge of the bent cap of pier *J24* near the side of the sculpture. According to the requirements of the *Technical Specification for Highway and Municipal Engineering Under Crossing High Speed Railway* (*TB 10182-2017*), during construction activities beneath a railway bridge, the impact on the horizontal and vertical displacement of bent caps should be limited to less than 2 mm. The maximum displacement of the bent caps is 1.78 mm, which meets the requirements of the regulations. The maximum settlement of the plain fill stratum is approximately 0.04 m, which is located at the base of sculpture *A*. After the construction of sculptures *A* and *B*, the ground settlement influence ranges connect, forming a saddle-shaped settlement tank. From Fig. 9, it can be observed that the piers are located outside the settlement tank, and the ground settlement is relatively small. Therefore, it can be inferred that the impact of ground settlement caused by sculpture construction on the piers is small. Taking all the above analysis into consideration, it can be concluded that the safe operation of the bridge can be ensured.

## Analysis of the internal force response of sculptures under strong wind loads

Apart from the pier structure's deformation induced by subsidence resulting from the sculpture's construction, the displacement of the sculpture itself and its potential to overturn due to high wind loads may also impact the bridge. To study the anti-overturning performance of the sculpture structure under the action of wind load and ensure the safety of train operation, deformation analysis of a sculpture structure in a flow field is carried out through computational fluid dynamics (CFD) simulation. The wind pressure resistance and anti-overturning performance of the sculpture structure are studied.

### Theoretical wind load calculation

To compare and analyze the correctness of the CFD numerical calculation, the theoretical calculation of wind load is carried out first. The average equivalent static wind load at any height *z* can be determined according to different site topographies, structure heights and structure shapes and is expressed by the following equation:

1 $$w_{zs} = \mu_{s} \mu_{z} w_{0}$$

where *w*_0_ is the reference wind pressure, *μ*_*s*_ is the wind load shape coefficient of the structure at height *z*, and *μ*_*z*_ is the height variation coefficient of wind pressure of the structure at height *z*.

According to the random vibration theory, the equivalent static wind load of the pulsating wind at any height *z* can be expressed by multiplying the equivalent static wind load at that position by the equivalent coefficient *η*_*z*_, as shown in the following equation:

2 $$w_{zd} = \eta_{z} \mu_{s} \mu_{z} w_{0}$$

Therefore, the superposition of the two is the equivalent static wind load at any height:

3 $$w_{z} = w_{zs} + w_{zd} = (1 + \eta_{z} )\mu_{s} \mu_{z} w_{0} = \beta_{z} \mu_{s} \mu_{z} w_{0}$$

where *β*_*z*_ is the wind vibration coefficient, which is the equivalent coefficient considering the action of pulsating wind.

Therefore, the equivalent static wind load at any height is obtained by multiplying the average equivalent static wind load by the wind vibration coefficient in the Chinese load code. The wind load shape coefficient *μ*_*s*_, the height variation coefficient *μ*_*z*_ and the reference wind pressure *w*_0_ in the equation can be determined with the current load code according to the structure type of the calculation object^31^. Since the wind speed is a constant value in this numerical calculation, *β*_*z*_ is taken as 1. According to the code requirements, when considering the seaside environment of the structure and a height of 15 m from sea level, the value of *μ*_*z*_ is 1.42. When the cross section of the structure is a rectangular plane, the value of *μ*_*s*_ is 0.8. The reference wind pressure is based on the wind pressure value of the 50-year average wind speed at a height of 10 m under the standard landform.

According to the size of sculpture *A*, the second highest structure A*2 (15 m) is taken as an example. Considering the altitude difference, it is basically equivalent to the 15 m specified in the code. Substituting each value into Eq. (3), the calculated wind load *w*_*z*_ at the top of sculpture A*2 is 0.38 MPa. Notably, this result is the calculated value of the theoretical formula of the single sculpture structure A*2. Because of the actual situation, sculpture *A* is a combination of multiple structures. Under the action of wind load, different structures will interact with each other. Therefore, there will be a deviation between the numerical calculation value and the theoretical calculation value.

### CFD numerical model construction

For this project, the stress and deformation response of the sculpture structure under the action of wind load is a fluid–solid coupling problem. The physical process is as follows: the air flows in the model, the sculpture structure acts as an obstacle to hinder the airflow, and the airflow affects the outer surface of the sculpture. Forces created by viscous resistance and masonry pressure are applied, and the sculpture deforms under the external load, thereby causing the airflow to follow a new path. Therefore, in this fluid–solid interaction problem, the sculpture structure is used as a deformable material to evaluate the alignment resistance against wind pressure and overturning.

Among the three sculptures, sculptures *A* and *B* are large in overall size and volume, and their impact area is also large. In addition, these sculptures are relatively close to piers *J23* and *J24*. Therefore, sculptures *A* and *B* are great potential safety hazards after construction is completed, so sculptures *A* and *B* are selected as the evaluation objects. The calculation is divided into two working conditions: (1) in working condition I, the wind load is loaded along the direction of the mid-span connection line of the sculpture structure; and (2) in working condition II, the wind load is loaded along the direction of the vertical mid-span connection line of the sculpture structure. The stress and deformation responses under two wind load conditions are simulated and analyzed by CFD software *COMSOL Multiphysics*, which is a versatile software for directly coupling analysis of multiphysics. *COMSOL Multiphysics* is a simulation software based on the finite element method (FEM), which employs the solution of partial differential equations or systems of partial differential equations to replicate real-world physical phenomena. According to the structural design drawings of sculptures *A* and *B*, the dimensional and shape contour information was extracted from the front view, side view, and top view. Firstly, 3D solid models of the sculpture structures are created using *Rhino* software. Then, they are exported as *.stl* files and imported into *COMSOL Multiphysics* software for calculation. To simulate the flow field environment, a hexahedral space with a size of 40 m × 40 m × 40 m is established outside the sculpture and divided into meshes.

### Calculation result of working condition I

#### Calculation result of sculpture A

**Figure 10:**
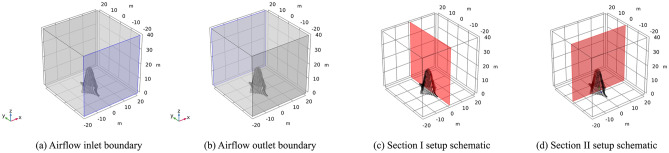
Working condition I of sculpture *A.*

CFD simulations involve both laminar flow and solid mechanics physics. For the laminar flow process, the numerical simulation process sets the inlet and outlet as boundary conditions. According to the bridge design documents, the wind speed for strong winds based on historical statistical data with a return period of 50 years is 23 m/s. Therefore, the strong wind speed is selected as 23 m/s (level 9 gale); that is, the inlet maintains an airflow of 23 m/s. The outlet is open to the atmosphere, and the corresponding section is set, as shown in Fig. 10.

The study presents a calculation of the wind load airflow velocity field, displacement, stress, and moment of section I and section II of the sculpture structures. The wind load is loaded along the direction of the mid-span connection line of the sculpture structure. The maximum airflow velocity at section I is approximately 27.5 m/s, located at the top of the sculpture structure. Meanwhile, the maximum displacement at section I is approximately 0.52 mm, also located at the top of the sculpture structure. The maximum stress of approximately 0.43 MPa occurs at the top of the sculpture A*2 structure. Additionally, the maximum moment of approximately 8 kN·m occurs at the top of the sculpture A*3 structure.

**Figure 11:**
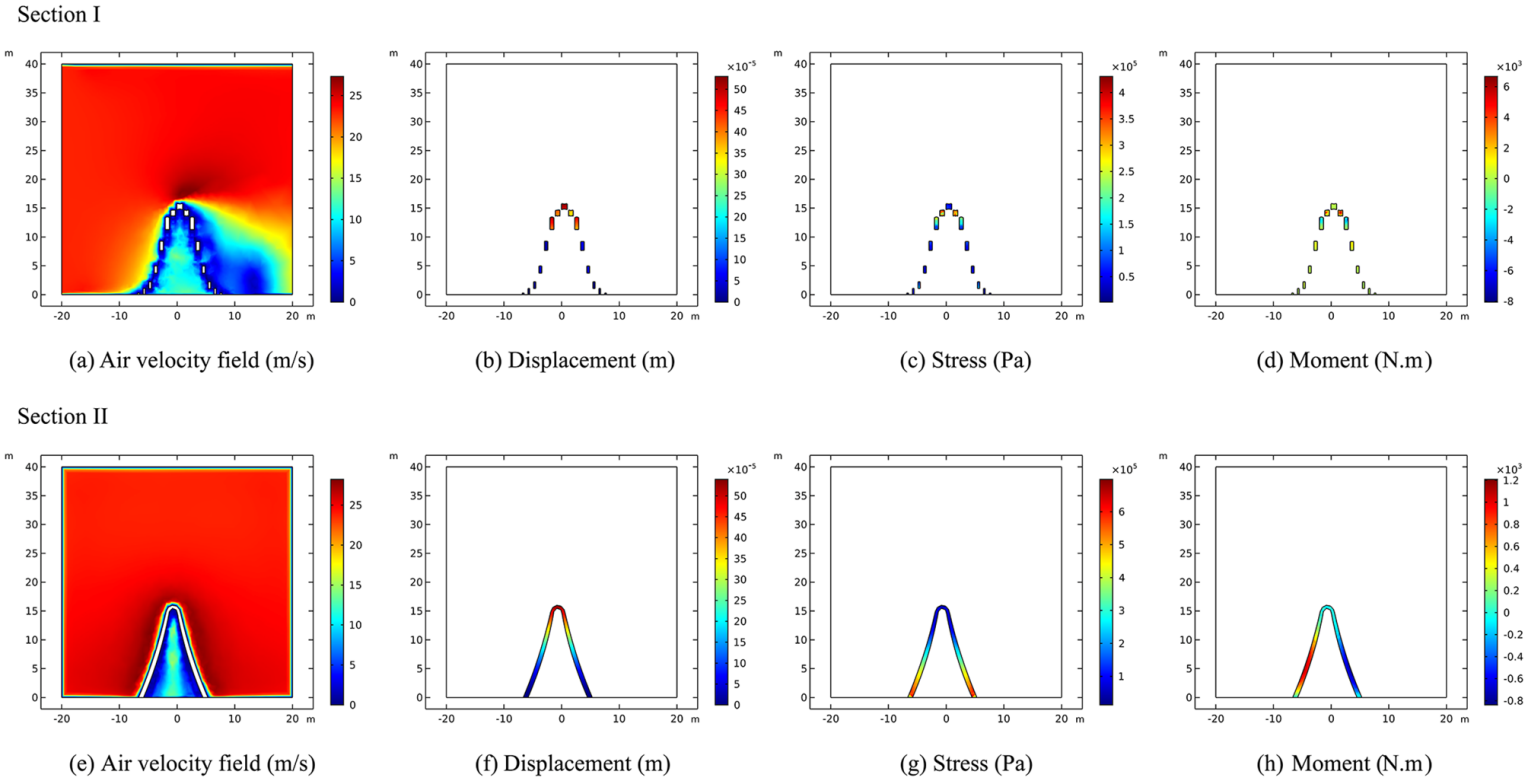
Calculation results of sculpture *A* in working condition I.

Similarly, when the wind load is loaded in the direction of the mid-span connecting line of the sculpture structure, the maximum airflow velocity at section II is approximately 27.5 m/s, located at the sculpture A*1 structure. The maximum displacement is approximately 0.53 mm, located at the top of the sculpture structure. The maximum stress is approximately 0.7 MPa, located at the bottom of the sculpture A*1 structure. Moreover, the maximum moment of approximately 1.2 kN·m is located above the foot of the sculpture structure, and the sculpture structure is in a torsion state. The calculation results are shown in Fig. 11.

#### Calculation result of sculpture B

**Figure 12:**
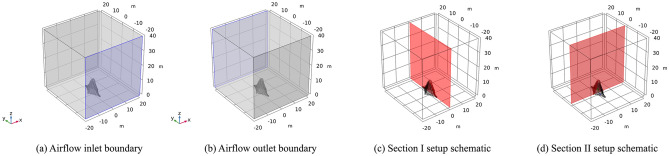
Working condition I of sculpture *B.*

In the numerical simulation process, the inlet and outlet are set as boundary conditions, and the strong wind speed is set the same as sculpture* A*. That is, the inlet maintains an airflow of 23 m/s, the outlet is connected to the atmosphere, and the corresponding section is set, as shown in Fig. 12.

The results indicate that the maximum airflow, with a velocity of approximately 27 m/s, is located at the top of the sculpture structure when the wind load is applied in the direction of the mid-span connecting line. The maximum displacement is approximately 0.45 mm, and the maximum stress is approximately 0.22 MPa. The maximum moment is approximately 1.2 kN·m, all of which are located at the top of the sculpture structure.

**Figure 13:**
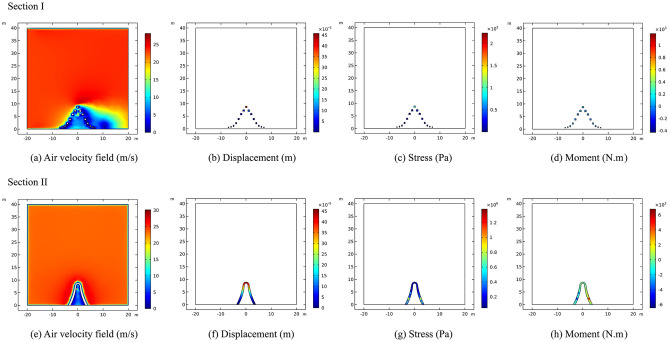
Calculation results of sculpture *B* in working condition I.

In section II, under wind load along the mid-span connecting line of the sculpture structure, the maximum airflow velocity at approximately 30 m/s is observed at sculpture B*1. The maximum displacement of approximately 0.45 mm occurs at the top of the sculpture structure. The maximum stress of approximately 1.4 MPa is observed at the middle of the sculpture B*1 structure. The maximum moment of approximately 6.5 kN·m is located above the feet of the sculpture structure, with opposite directions on the two sides, indicating a torsion state of the sculpture structure. The calculation results are shown in Fig. 13.

It can be seen that the sculpture structure has a certain obstruction effect on the wind flow. Due to the large size of sculpture* A*, there is a certain space between its components, which provides a pathway for the transmission of wind flow. Therefore, the wind speed can reach 10–15 m/s under the sculpture A*1 structure. Moreover, when the wind flow encounters obstruction from the sculpture structure, the wind speed drops sharply to near 0 m/s near the structure, resulting in higher displacement and internal forces. As for sculptures* B*, due to its small size, its obstruction effect on the wind flow is very obvious. The wind speed below the sculpture structure is almost all below 10 m/s.

### Calculation result of working condition II

#### Calculation result of sculpture A

**Figure 14:**
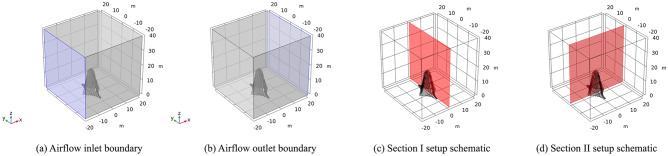
Working condition II sculpture *A.*

During the numerical simulation process of working condition II, the inlet and outlet are also subjected to boundary conditions. Similarly, a wind load of 23 m/s (level 9 gale) wind speed is still selected. Specifically, the inlet was maintained at a constant airflow, while the outlet was connected to the atmosphere with a corresponding cross-section, as illustrated in Fig. 14.

Section I experiences the wind load in the direction of the vertical mid-span connecting line of the sculpture structure. The results indicate that the maximum airflow velocity of approximately 31 m/s is located at the edge of each structure of the sculpture. The maximum displacement of approximately 0.48 mm is located at the top of the sculpture A*3 structure. The maximum stress of approximately 3 MPa is also located at the top of the sculpture A*3 structure. The maximum moment of approximately 4.5 kN·m is located at the top of the sculpture A*3 structure, with the moment directions of the two structures being opposite.

**Figure 15:**
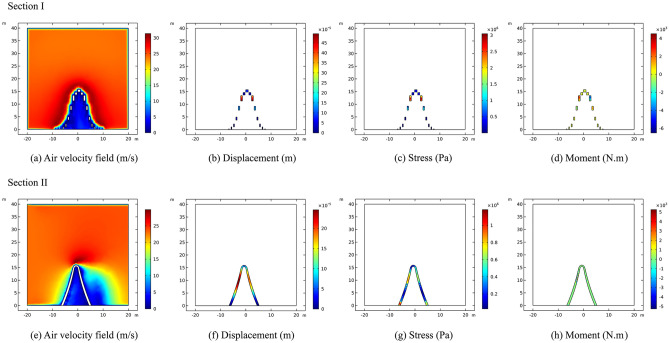
Calculation results of sculpture *A* in working condition II.

In section II, the wind load is loaded in the direction of the mid-span connecting line of the sculpture structure. The maximum airflow velocity of approximately 29 m/s is located at the top of the sculpture. The maximum displacement of approximately 0.33 mm is located on the windward side. The maximum stress of approximately 1.2 MPa is located on the windward side of the bottom of the sculpture. The maximum moment of approximately 5.2 kN·m is located on the windward side above the middle of the sculpture structure. The calculation results are presented in Fig. 15.

#### Calculation result of sculpture B

**Figure 16:**
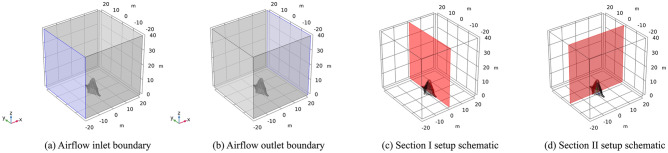
Working condition II of sculpture *B.*

The boundary conditions of the numerical simulation of *sculptures B* are the same as before, and a set of airflow inlet and outlet boundaries are also determined, as shown in Fig. 16.

For section I, the wind load is applied in the direction of the vertical mid-span connection line of the sculpture structure, and the maximum airflow is approximately 27.5 m/s, which occurs at the edge of each structure of the sculpture. The maximum displacement is approximately 0.05 mm, which is located at the top of the sculpture. The maximum stress is approximately 0.3 MPa, which is located at the top of the sculpture B*1 structure. The maximum moment is approximately 1.2 kN·m, which is also located at the top of the sculpture B*1 structure.

**Figure 17:**
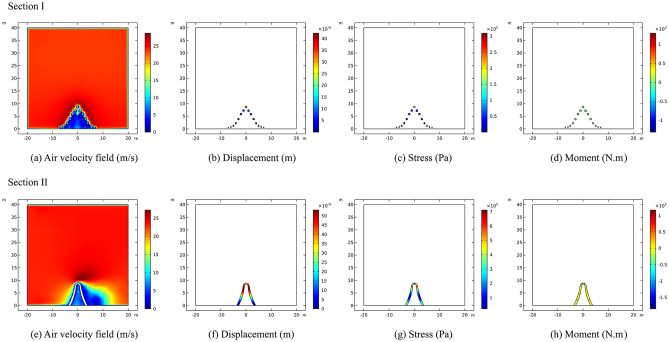
Calculation results of sculpture *B* in working condition II.

In Section II, the calculation results show that the maximum airflow velocity is approximately 26 m/s, located at the top of the sculpture. The maximum displacement is approximately 0.05 mm, occurring in the middle and upper parts of the sculpture structure. The maximum stress is approximately 0.7 MPa, located at the upper part of the sculpture structure. The maximum moment is approximately 1.1 kN·m, located on the windward side above the sculpture structure. The calculation results are illustrated in Fig. 17.

Unlike working condition I, due to the wind direction being perpendicular to the front face of the sculpture structure, the wind flow is hindered by a larger area when the spacing between the sculpture components is small. Therefore, regardless of section I or section II, the wind speed inside the space below the sculpture is severely hindered and reduced to near 0 m/s. Compared with condition I, the stress value of sculptures *A* is significantly increased, while the deformation of sculptures *B* is significantly reduced. The main reason is that the influence of wind direction change on sculptures *A* is greater, while the smaller size of sculptures *B* weakens the influence of wind direction change. According to the mechanism of fluid–structure interaction, when the wind load acts on the solid surface, the more the wind speed decreases, the greater the internal force of the solid will be. Hence, even under extreme conditions as stipulated, the construction of the irregular sculpture structure group described in this paper does not display any discernible tendency towards toppling. In light of these circumstances, both the bridge structure and the sculpture structures are able to ensure a reasonable level of safety.

### Comparative analysis

#### Comparative analysis of theoretical calculation and CFD calculation

To verify the accuracy of the CFD numerical simulation results, a comparative analysis was conducted with the theoretical calculation results obtained in section “Theoretical wind load calculation”. According to the theoretical calculations, the wind pressure acting on the top of A*2 is approximately 0.38 MPa. This calculation result represents the stress value on the structure's surface when the wind load is applied to the front of the sculpture. In the CFD calculation results (section “Calculation result of sculpture A”), the corresponding value is approximately 0.43 MPa. The difference between the two values is about 11%. Considering the simplifications made in the calculations, the calculation results of the two are basically consistent.

#### Comparative analysis of CFD calculation in two different working conditions

To analyze and compare the internal force responses of two different wind directions on typical sculpture structures, the largest structures of sculpture *A* and sculpture *B*, namely, the sculpture A*1 structure and sculpture B*1 structure, were selected for displacement, stress, and moment analysis under the two different working conditions. It should be noted that the internal forces on the outer contour lines of the two sculptures' structures at section II were observed, instead of the entire section. This leads to slight differences in the numerical analysis of internal forces compared to the previous analysis of the entire section.

**Figure 18:**
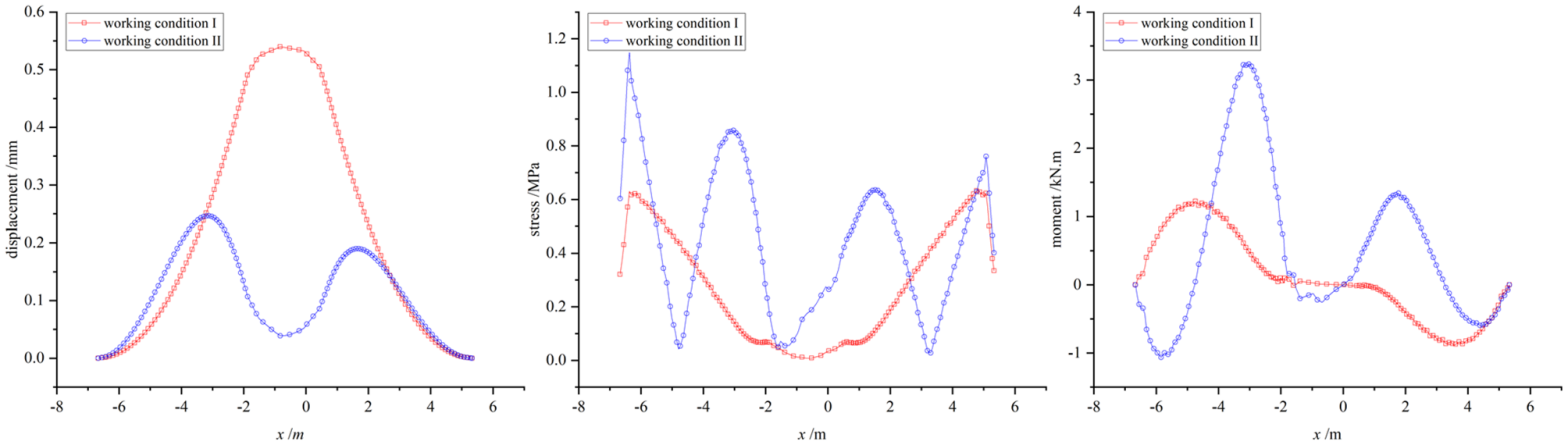
Internal forces of sculpture A*1 structure.

From Fig. 18, it can be seen that for the two different wind directions, except for displacement, the stress and bending moment values of the sculpture A*1 structure under working condition I are generally smaller than those under operating condition II. Under working condition I, due to the wind direction being perpendicular to the front of the structure, the displacement distribution shows a clear pattern of being smaller at the bottom and larger at the top. Under working condition II, the maximum displacement occurs at the middle of the structure, and the displacement sizes on the windward and leeward sides are similar. Regarding stress, when the wind direction is frontal, the stress value at the bottom is significantly greater than that at the top, which is due to the strong restraining and fixing effect of the shallow-buried pile foundation on the sculpture structure, while the top is the free end. This stress response is consistent with the displacement response. Under working condition II, the stress distribution of the sculpture structure shows an oscillating pattern, and the stress value on the windward side is higher than that on the leeward side. The distribution of bending moment is greatly affected by the wind direction. Under working condition I, the moment is symmetric about the sculpture structure, resulting in the opposite sign of the moment. Under working condition II, the moment on the windward side is generally higher than that on the leeward side, and the maximum value appears in the upper part of the windward side.

**Figure 19:**
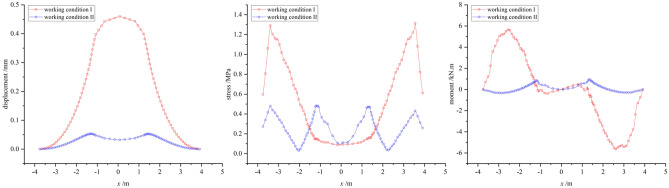
Internal forces of sculpture B*1 structure.

In contrast to sculpture A*1 structure, the displacement, stress, and moment of sculpture B*1 structure in working condition I are all larger than those in working condition II, as shown in Fig. 19. In working condition I, the overall distribution pattern of displacement, stress, and moment is similar to that of sculpture A*1 structure. In working condition II, when the wind direction is from the side, the oscillation of the internal force distribution is not strong. Due to its smaller size and the influence of other sculpture structures, the response of moment is weaker under wind loads. Therefore, it can be seen that the size has a significant impact on the internal forces of the sculpture structures when the wind direction is fixed.

## Conclusions

By conducting calculation and analysis on the construction of the sculpture structure and its response to wind loads, the following conclusions can be drawn:

1. The construction of the sculptures has a minor impact on the safety of the bridge structure. The maximum horizontal displacement of the bridge pier is approximately 1.78 mm, which occurs at the edge of the bent cap of pier *J24* near the side of the sculpture, and the maximum vertical displacement is approximately 0.54 mm, which occurs at the edge of the bent cap of pier *J24* near the side of the sculpture.
2. When the wind load is loaded in the direction of the vertical mid-span connection line of the sculpture structure, the structural deformation response of sculpture *A* on the windward side is higher, except for the moment, which is an unfavorable wind direction. When the wind load is loaded in the direction of the mid-span connection line of the sculpture structure, the deformation response of the sculpture *B* structure is higher than that when the wind load is loaded in the direction of the vertical mid-span connection, which is an unfavorable wind direction. The sculptures are relatively stable under both conditions.
3. Under working condition I, the displacement is smaller at the bottom and larger at the top. The stress distribution on the windward side is higher than that on the leeward side under working condition II. The bending moment distribution is affected by the wind direction, with symmetric moments under working condition I and higher moments on the windward side under working condition II. The size of the sculpture structures also influences their internal forces under fixed wind directions.

## Data Availability

The datasets used and analysed during the current study available from the corresponding author on reasonable request.
